# Cross-talk between the gut microbiota and hypothyroidism: a bidirectional two-sample Mendelian randomization study

**DOI:** 10.3389/fnut.2024.1286593

**Published:** 2024-03-18

**Authors:** Chao Shi, Jie Chen, Siying He, Yingying Zhang, Yanyue Zhang, Lisha Yu

**Affiliations:** Department of Laboratory, Jinhua Central Hospital, Zhejiang, Jinhua, China

**Keywords:** hypothyroidism, gut microbiome, causal effect, Mendelian randomization, probiotics

## Abstract

**Background:**

Multiple observational studies suggest a connection between the composition of the gut microbiota and hypothyroidism. However, it has yet to be determined whether the gut microbiota has a causal effect on hypothyroidism.

**Methods:**

To investigate the connection between the gut microbiota and hypothyroidism, two-sample Mendelian randomization was performed using data from a genome-wide association study meta-analysis (*n* = 18,430) conducted by the MiBioGen consortium. Summary statistics for hypothyroidism (26,342 cases and 59,827 controls) were obtained using the data from the FinnGen consortium R8 release data. To investigate the causal link between the gut microbiota and hypothyroidism, various methods, including MR-Egger, weighted median, weighted model, simple model, MR-PRESSO, and inverse variance weighted (IVW), were employed. The bacteria that were causally linked to hypothyroidism in forward Mendelian randomization analysis were subjected to reverse Mendelian randomization analysis. Cochran’s *Q* statistics were utilized to gauge the heterogeneity of the instrumental variables.

**Results:**

The results indicated that Akkermansia had a positive impact on hypothyroidism, with an odds ratio of 0.84 (95% CI 0.74–0.95, *p* = 0.01) based on the inverse variance-weighted estimates. Additionally, Anaerostipes (OR = 1.17, 95% CI 1.01–1.36, *p* = 0.04), Butyrivibrio (OR = 0.93, 95% CI 0.88–0.99, *p* = 0.02), Holdemania (OR = 0.89, 95% CI 0.81–0.99, *p* = 0.03), Intestinimonas (OR = 1.13, 95% CI 1.02–1.26, *p* = 0.03), Ruminiclostridium5 (OR = 1.19, 95% CI 1.01–1.41, *p* = 0.04), and Ruminococcaceae UCG-011 (OR = 0.91, 95% CI 0.84–0.99, *p* = 0.03) were identified. The gut microbiota was not significantly affected by hypothyroidism, as indicated by the results of the reverse MR analysis. There was no significant variation in the instrumental variables or horizontal pleiotropy.

**Conclusion:**

The findings of this study using two-sample Mendelian randomization indicate a causal relationship between Akkermansia and hypothyroidism. Increased Akkermansia inhibits the onset and progression of hypothyroidism. Additional randomized controlled experiments are necessary to elucidate the beneficial impact of probiotics on hypothyroidism and their distinct protective mechanisms.

## 1 Introduction

Hypothyroidism, which is a hormonal imbalance caused by reduced functioning of the thyroid gland and an inadequate production of thyroid hormones, is a prevalent global ailment (1). Worldwide, environmental iodine deficiency is the predominant factor causing thyroid disorders, such as hypothyroidism, while autoimmune thyroiditis is the main cause of primary hypothyroidism in areas with adequate iodine levels (2). Hypothyroidism typically has a gradual onset and can initially be difficult to diagnose due to its vague and nonspecific symptoms. According to a prevalence study conducted in Europe, approximately 5% of the population is affected by hypothyroidism, with an additional 5% potentially having an undiagnosed thyroid disorder (3, 4). Various health complications, including heart disease, infertility, and impaired brain development in children, can arise as a result of hypothyroidism (5–7). Currently, the approach to managing hypothyroidism involves alleviating symptoms and minimizing additional damage. However, this condition places a significant burden on both the economy and the quality of life for those affected (8, 9). As a result, a thorough investigation into the underlying causes and the development of new treatment alternatives are vital.

The gut microbiota is now considered a key element in regulating host health. Currently, imbalances in the gut microbiota are associated with numerous ailments, such as overweight, type 2 diabetes, fatty liver disease, inflammatory bowel diseases (IBDs), and various forms of cancer (10–12). Over the past few years, an increasing number of research investigations have been carried out on the relationship between the thyroid and the intestines. Gut and thyroid follicular cells have some morphological and functional similarities due to their common embryological origin (13, 14). Alterations in the intestinal microbiota can indirectly impact thyroid function. Simultaneously, numerous research investigations have examined alterations in the gut microbiota among individuals diagnosed with hypothyroidism. 16S rRNA gene sequencing was used to conduct metagenomic analysis on fecal samples from a rat model of hypothyroidism (caused by propylthiouracil or thyroidectomy). The analysis showed a noteworthy decrease in Prevotella within the hypothyroid group compared to the normal group (15). According to a clinical trial, probiotic supplementation did not significantly impact thyroid-stimulating hormone (TSH) levels but improved well-being in individuals with hypothyroidism (16). Simo Liu’s research also unveiled a notable surge in Phascolarctobacterium among hypothyroid participants. Comparatively, patients with HT exhibited significantly lower bacterial richness and diversity compared to the control group, particularly in cases of hypothyroidism (17). Nevertheless, the precise correlation between the gut microbiota and hypothyroidism remains uncertain. Historical research has predominantly consisted of case-control studies, making it challenging to ascertain the timing and impact of exposure on outcomes. Moreover, age, environment, diet, and lifestyle are potential confounding factors in observational studies that may affect the relationship between the gut microbiota and hypothyroidism.

The utilization of Mendelian randomization (MR) in this context presents a new method for investigating the causal link between the gut microbiota and hypothyroidism. Unlike conventional observational studies, MR analysis can eliminate the bias of reverse causation because allelic randomization always occurs before the onset of the disease (18). MR analysis can reduce confounding factors by incorporating genetic markers as instrumental variables (IVs) through random segregation and an independent assortment of genetic polymorphisms during conception (19, 20). Causality exploration is made possible by large-scale genome-wide association studies (GWAS) (21). To assess the causal relationship between the gut microbiota and hypothyroidism, a two-sample MR analysis was conducted using summary statistics from GWAS conducted by the MiBioGen and FinnGen consortia.

## 2 Methods

### 2.1 Study design and the assumption of MR

**Figure 1:**
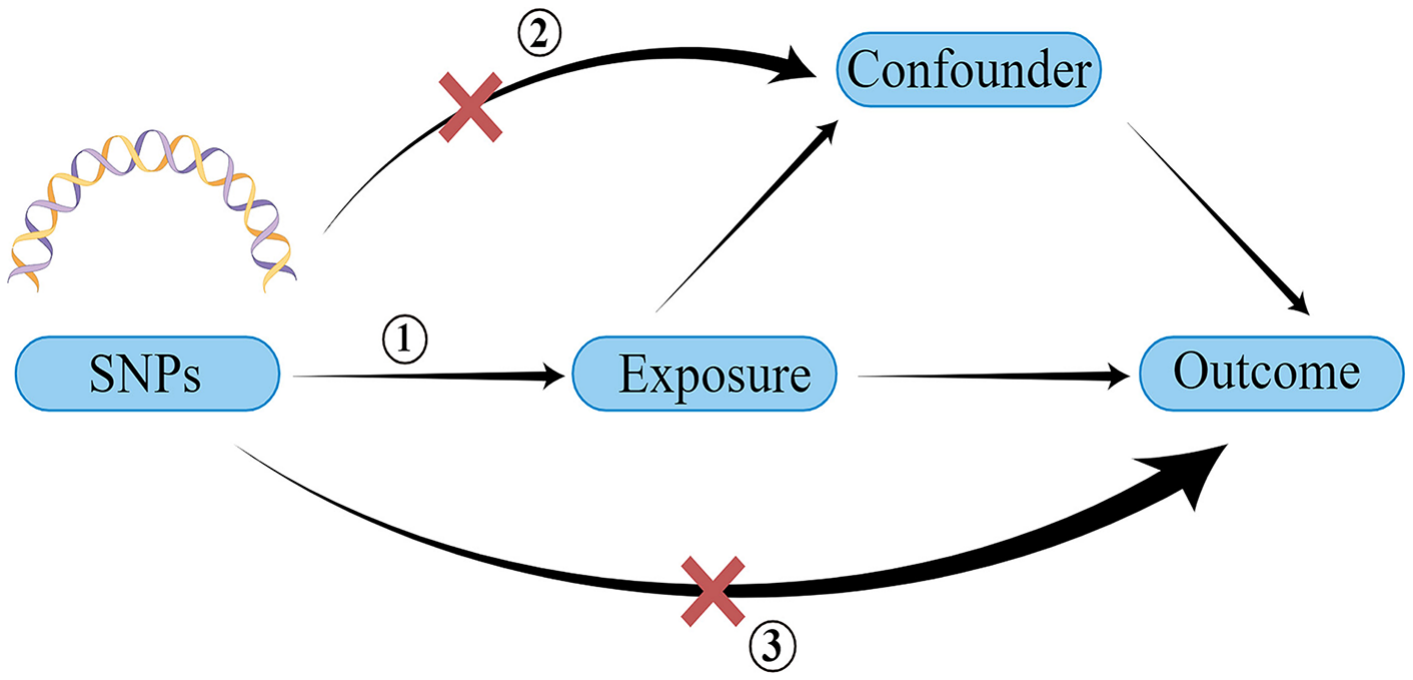
Schematic of the three core assumptions of Mendelian randomization research. SNP, single nucleotide polymorphism. Created by Figdraw.com.

A schematic representation of the study design and the three core assumptions of MR are shown in Figure 1 and are as follows: (1) a strong association exists between single nucleotide polymorphisms (SNPs) and exposure; (2) known confounders do not influence SNPs; and (3) SNPs solely impact the outcome through exposure (22).

### 2.2 Data sources

Instrumental variables (IVs) were chosen from a GWAS dataset of the international MiBioGen consortium, specifically targeting SNPs associated with the composition of the human gut microbiome (23, 24). The study involved a diverse and extensive GWAS that combined the analysis of 16S ribosomal RNA gene sequencing profiles and genotyping data from a total of 18,340 individuals across 24 cohorts from various countries, including the United States, Canada, Israel, South Korea, Germany, Denmark, the Netherlands, Belgium, Sweden, Finland, and the United Kingdom. The study aimed to analyze the variable regions V4, V3–V4, and V1–V2 of the 16S rRNA gene to evaluate the microbial composition and conduct taxonomic classification using direct taxonomic binning. All datasets were normalized to 10,000 reads per sample to address variations in sequencing depth. Microbiota quantitative trait loci (mbQTL) mapping analysis was utilized to identify host genetic variants associated with specific genetic loci linked to the abundance levels of bacterial taxa in the gut microbiota. The ultimate dataset consisted of 211 species, encompassing 131 genera, 35 families, 20 orders, 16 classes, and 9 phyla. During the study, the genus was considered the most basic level of taxonomy, and 131 genera were identified with an average abundance exceeding 1%, among which 12 genera remained unidentified (23). Hence, the analysis of the present study involved a total of 119 taxa at the genus level. The summary data for hypothyroidism in the GWAS were acquired from the analysis of the FinnGen consortium R8 release (25), comprising 86,169 samples (26,342 cases and 59,827 controls) and encompassing a dataset of 16,378,441 SNPs.

### 2.3 Instrumental variables

During the process of screening for valid IVs, we conducted a thorough review of published articles pertaining to Mendelian randomization (26, 27). To choose SNPs as IVs that may suggest a potential link between the gut microbiome and hypothyroidism, various thresholds were established according to variations in exposure. When the gut microbiome was considered as the exposure, our initial selection of instrumental variables involved choosing SNPs that fell below the genome-wide statistical significance threshold of 5 × 10^−8^. Regrettably, this approach yielded only a limited number of gut microbiota as IVs. To obtain a more comprehensive understanding of the causal relationship between hypothyroidism and the gut microbiota, we subsequently implemented a secondary threshold, specifically identifying SNPs below the locus-wide significance level of 1 × 10^−5^. Upon selecting hypothyroidism as the exposure, the significance level of the IVs was determined by the genome-wide statistical threshold (*p* < 5 × 10^−8^). Pairwise linkage disequilibrium was used to assess the independence of the selected SNPs. If *R*^2^ was greater than 0.001 (with a clumping window of 10,000 kb), the SNP that had a higher *p*-value or was correlated with more SNPs was removed. Simultaneously, SNPs with a minor allele frequency (MAF) of ≤0.01 were eliminated (28). The *F*-statistic was computed using the equation *F* = *R*^2^ × (*N* − 1 − *K*)/(1 − *R*^2^) × *K* to assess the potency of individual SNPs. Here, *R*^2^ denotes the proportion of variability in the exposure elucidated by the genetic variants, *N* signifies the sample size, and *K* indicates the count of instruments. If the *F*-statistics exceeded ten, SNPs were deemed sufficiently strong to counteract any potential bias (29).

### 2.4 Statistical analysis

To investigate the potential causal relationship between the gut microbiota and hypothyroidism, this research employed various approaches, such as IVW (inverse variance weighted) (30), simple mode (31), MR-Egger regression (32), weighted median (33), and weighted model (32). The specific descriptions of each method are summarized in Table 1.

**Table 1:** Characteristics of five Mendelian randomization (MR) methods.

Method	Description	Reference
Inverse variance weighted	The conventional method of MR utilizes a meta-analysis technique to merge the wald ratio estimates of the causal effect derived from various SNPs. The estimates obtained from IVW MR represent a weighted linear regression of SNP-outcome links on SNP-exposure associations, with the intercept fixed at zero	(30)
MR-Egger	MR-Egger regression differs from IVW in that it does not require a slope through zero, allowing for a genotype-outcome dose–response relationship while accounting for pleiotropic effects. It does require the InSIDE assumption to hold, which means that the strength of the gene-exposure association should not correlate with the strength of bias due to pleiotropy	(32)
Weighted median	The weighted median method can still provide unbiased estimates even if half of the information comes from invalid IVs. Additionally, the weighted mode method can remain consistent even if the IVs are invalid as long as the valid instruments produce the highest number of similar individual instrument causal effect estimates	(33)
Weighted mode	If the majority of individual instrument causal effect estimates are valid, the weighted mode method remains consistent even if the IVs are invalid. In cases where the InSIDE hypothesis is violated, the weighted model estimate has been proven to be more effective in detecting a causal effect, with less bias and lower type I error rates as compared to the MR-Egger regression	(32)
Simple mode	The “simple mode” refers to an unweighted mode found in the empirical density function of causal estimation	(31)

IVs were evaluated for compatibility in sensitivity analyses by assessing heterogeneity. Heterogeneity was tested using Cochran’s *Q* statistics with the IVW and MR-Egger methods. A *Q* statistic exceeding the number of instruments minus one may serve as an indication of heterogeneity and the presence of invalid instruments. The significance of *Q* statistics at a *p* value less than 0.05 may also suggest the existence of heterogeneity. In cases where the *p* value exceeds 0.05 and no evidence of heterogeneity is found, the fixed-effects IVW method is considered the primary approach. Conversely, the random-effects IVW method was employed if substantial heterogeneity was observed. In situations where there is no excessive heterogeneity in the variant-specific causal estimates, the random-effects and fixed-effect outcomes will be identical, thereby ensuring no loss of precision (34, 35). The existence of horizontal pleiotropy suggests that IVs are associated with outcomes through mechanisms other than causal effects, which may lead to erroneous positive findings (*p* < 0.05). To assess the likely impact of horizontal pleiotropy, MR-PRESSO and MR-Egger regression tests were employed to examine the potential presence of horizontal pleiotropy. Statistical analyses were performed using MR-Egger’s intercept term (egger_intercept) and 0. If the *p* value exceeded 0.05, it was inferred that no horizontal pleiotropy was present (36, 37). SNPs were arranged in ascending order based on their MR-PRESSO outlier test *p* values and subsequently eliminated one by one. The MR-PRESSO global test was iteratively performed on the remaining SNPs by sequentially excluding each SNP from the list. This recursive procedure was repeated until the *p* value for the global test attained statistical insignificance. The MR-PRESSO outlier test yielded a *p* value denoting the significance of pleiotropy for each SNP, whereas the MR-PRESSO global test provided a *p* value representing the overall horizontal pleiotropy. A *p* value exceeding 0.05 indicates the results’ robustness and the reliability of causal inferences (36).

Furthermore, to determine if a single SNP significantly influences the causal impact of exposure on the outcome, a leave-one-out analysis should be conducted. The input for the leave-one-out analysis involved harmonized data from exposure and outcome, which were tested using the IVW method. To evaluate the causal connection between the gut microbiota and hypothyroidism, reverse MR analysis was conducted on bacteria that were identified as causally linked to hypothyroidism in the forward MR analysis. The techniques and configurations employed were in line with progressive MR.

**Figure 2:**
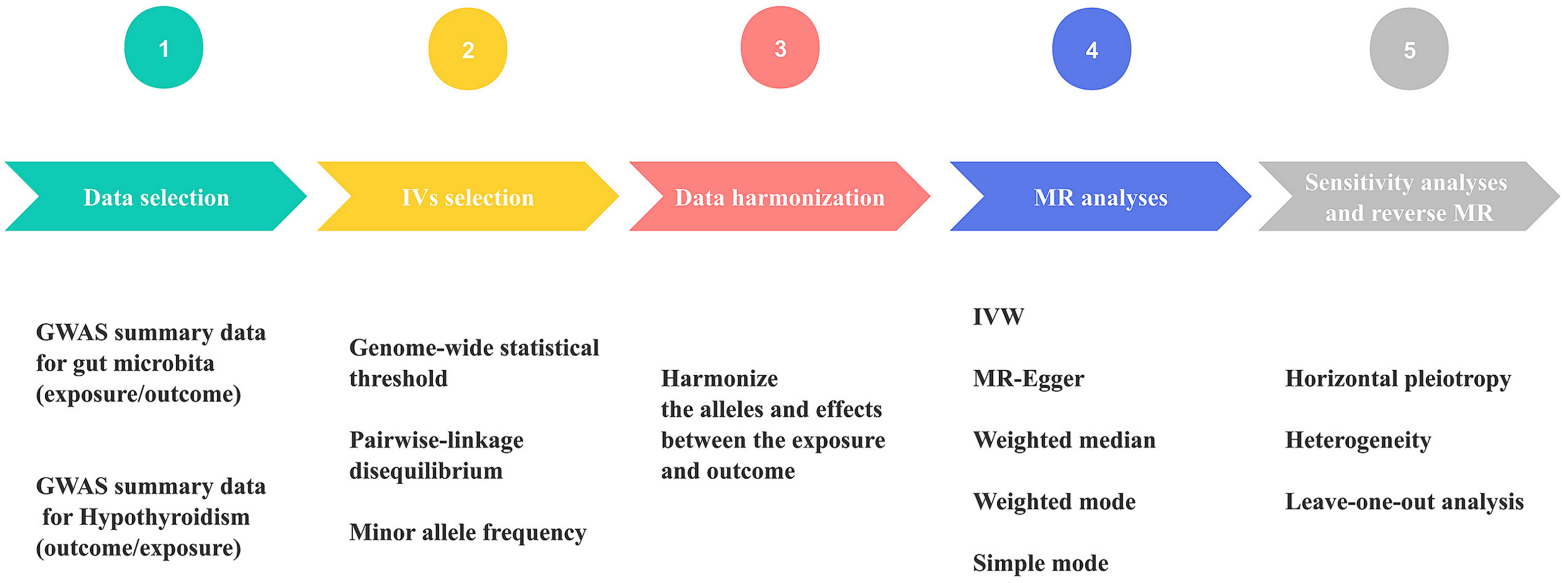
Schematic diagram of Mendelian randomization (MR) analysis process. The workflow was performed twice for gut microbiome and hypothyroidism (HY). GWAS, genome-wide association study; IVs, instrumental variables; IVW, inverse variance weighting; MR, Mendelian randomization.

All statistical analyses were conducted using R version 4.2.3 (R Foundation for Statistical Computing, Vienna, Austria). For the purpose of performing MR analyses, TwosampleMR (0.5.6) (38) and MR-PRESSO (1.0) (36) were employed. Figure 2 displays the MR diagram utilized in this study.

## 3 Results

A total of 119 bacterial genera were analyzed using 1,231 SNPs as IVs based on the IV selection criteria. Supplementary Table S1 details the selected instrumental variables. No weak instrument bias was observed, as the *F*-statistic for each SNP exceeded 10.

**Figure 3:**
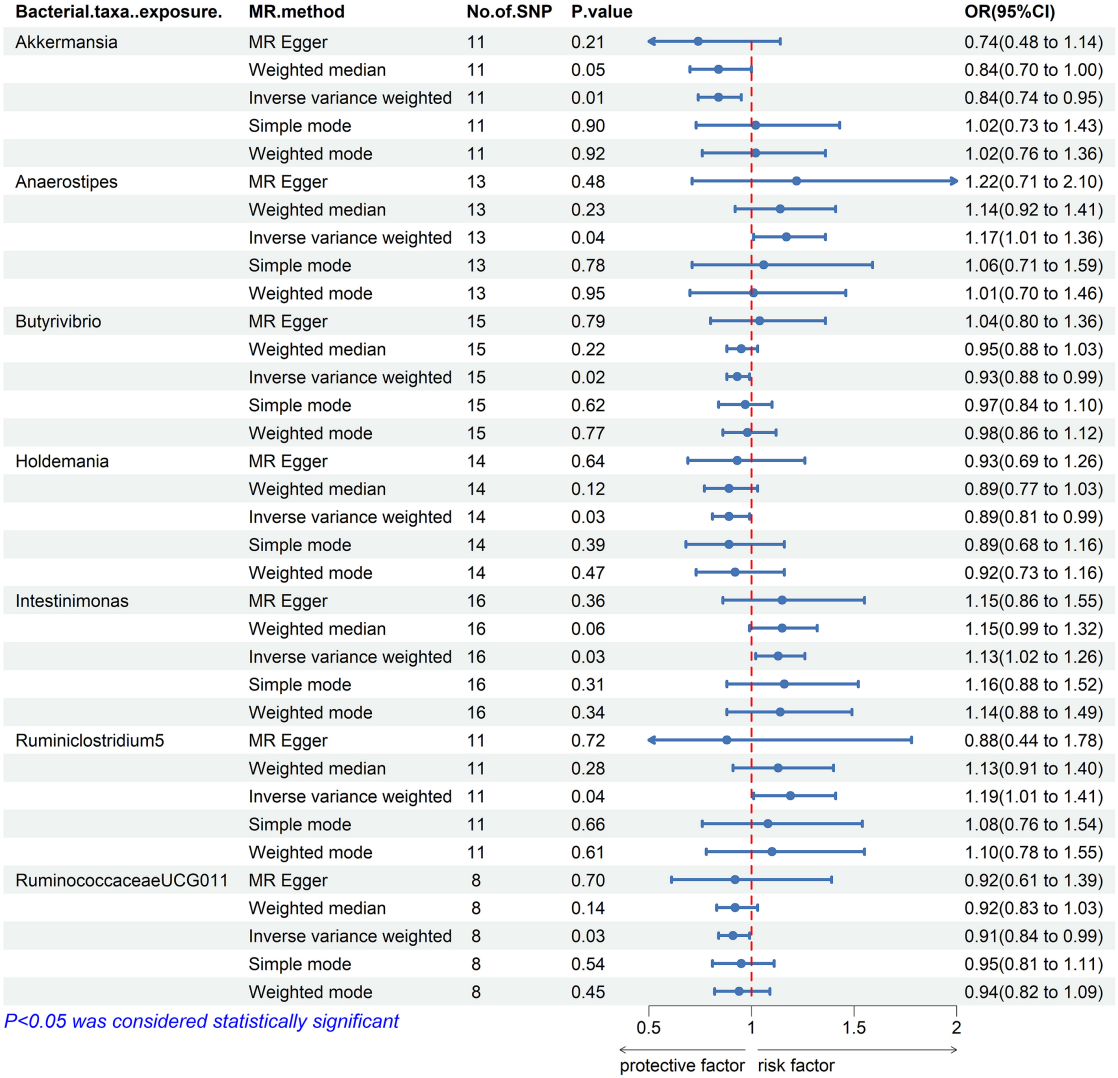
Mendelian randomization results of causal effects between the gut microbiome and Hypothyroidism. MR, Mendelian randomization; SNP, single nucleotide polymorphism; OR, odds ratio; CI, confidence interval.

**Figure 4:**
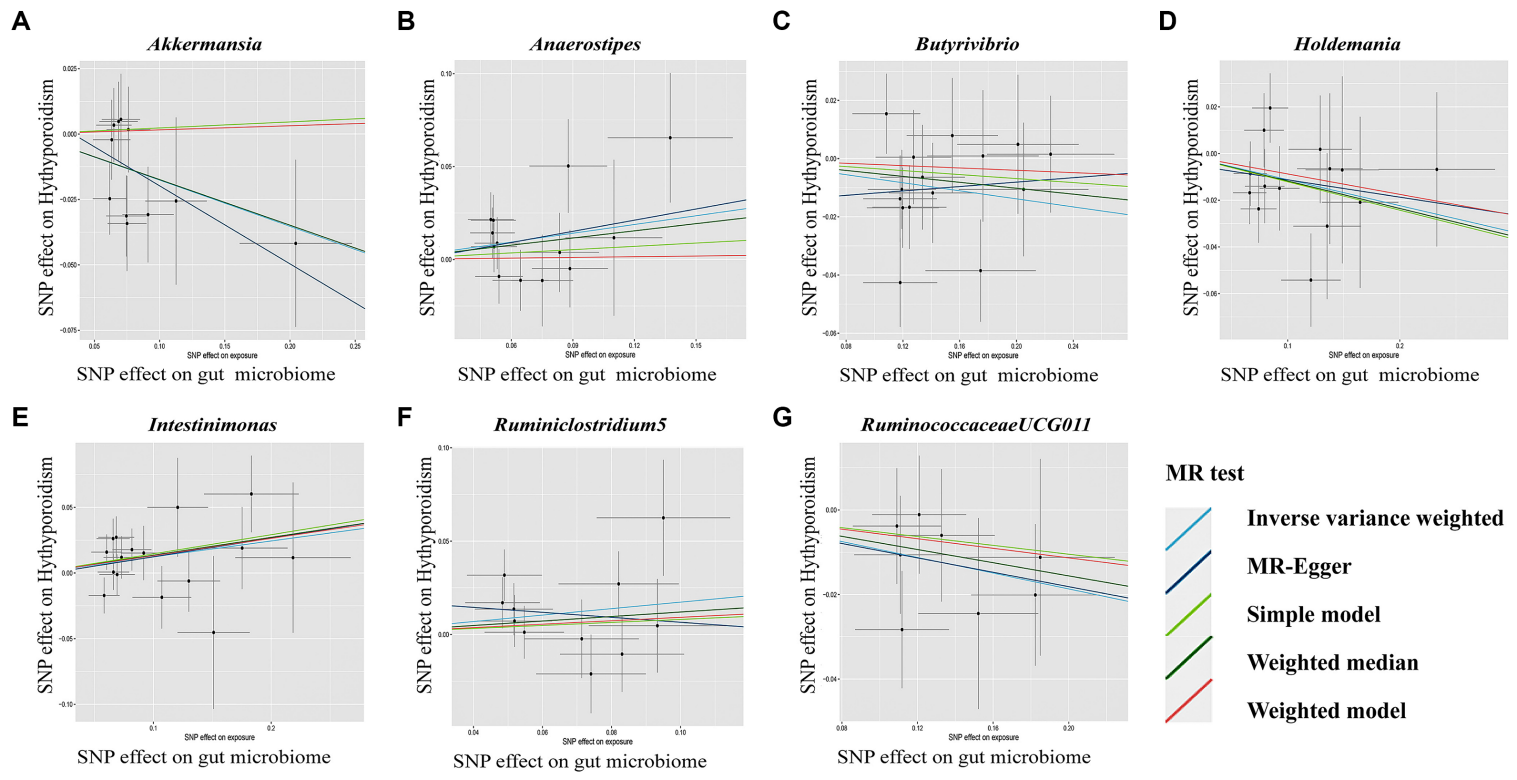
Scatterplot **(A-G)** of the effect of the gut microbiome on Hypothyroidism. In each plot, individual single nucleotide polymorphisms (SNPs) from the summary dataset of the gut microbiome genome-wide association study (GWAS) are represented by dots. The *x*-axis denotes the impact of these SNPs on the gut microbiome, while the *y*-axis signifies their influence on Hypothyroidism. Distinct colors of lines correspond to various Mendelian randomization (MR) methods. A positive slope indicates that exposure is a risk factor, whereas a negative slope signifies the contrary.

Our result displayed the presence of seven bacterial genera, namely, Akkermansia, Anaerostipes, Butyrivibrio, Holdemania, Intestinimonas, Ruminiclostridium5, and Ruminococcaceae UCG-011, which were linked to hypothyroidism in at least one MR method (Figures 3, 4). IVW estimates indicated that Akkermansia (OR = 0.84, 95% CI 0.74–0.95, *p* = 0.01), Ruminococcaceae UCG-011 (OR = 0.91, 95% CI 0.84–0.99, *p* = 0.03), Butyrivibrio (OR = 0.93, 95% CI 0.88–0.99, *p* = 0.02), and Holdemania (OR = 0.89, 95% CI 0.81–0.99, *p* = 0.03) exhibited a protective impact on hypothyroidism. Furthermore, Anaerostipes (odds ratio = 1.17, 95% CI 1.01–1.36, *p* = 0.04), Intestinimonas (odds ratio = 1.13, 95% CI 1.02–1.26, *p* = 0.03), and Ruminiclostridium5 (odds ratio = 1.19, 95% CI 1.01–1.41, *p* = 0.04) grow differentially, adding a detrimental effect to hypothyroidism. Moreover, the bacteria that were causally linked to hypothyroidism in forward MR analysis were subjected to reverse MR analysis. Hypothyroidism and the gut microbiota were not causally associated (Supplementary Tables S5–S8; Supplementary Figure 1).

**Figure 5:**
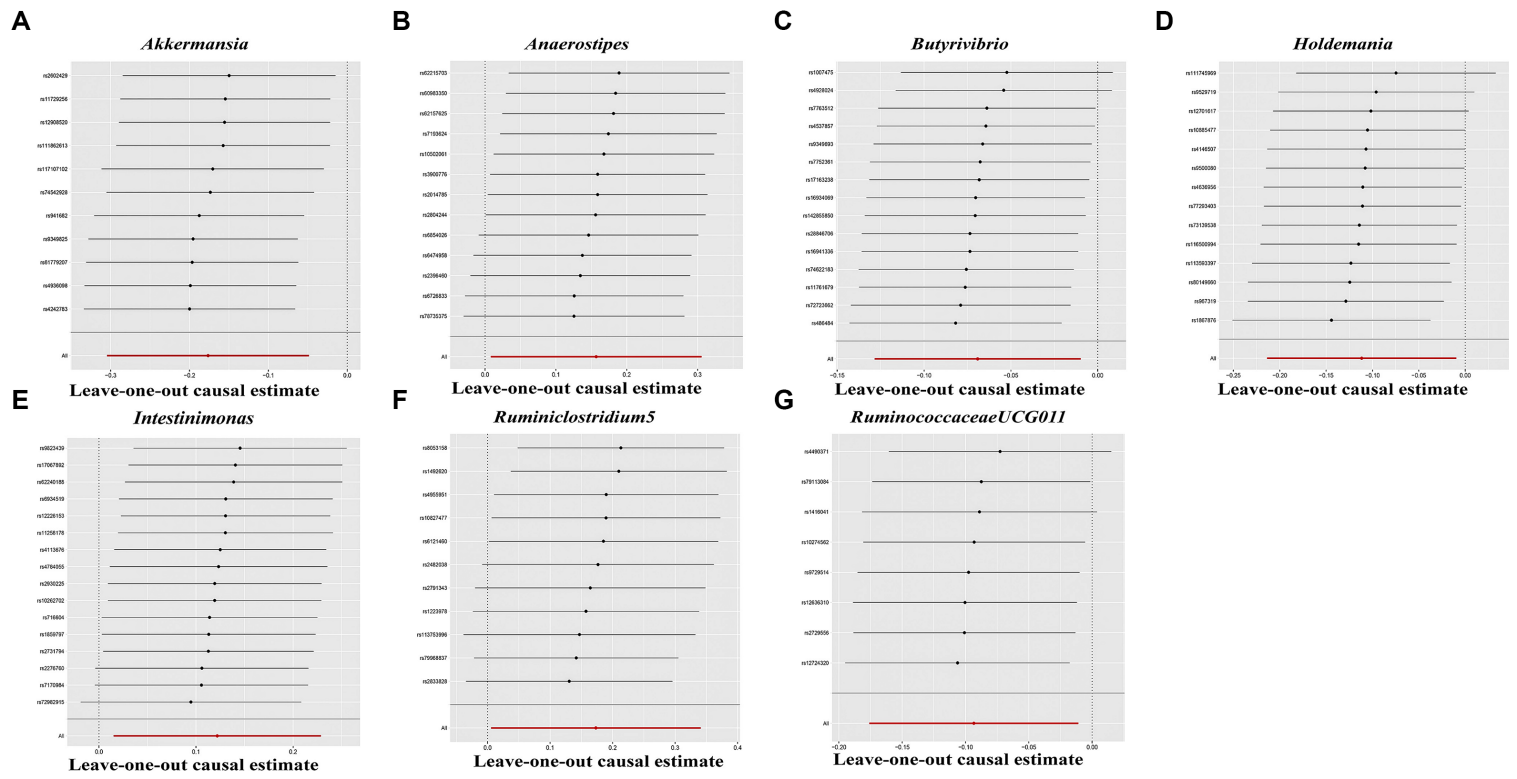
Leave-one-out analysis for gut microbiome on Hypothyroidism. The sensitivity of the causal effect was assessed across different components **(A–G)** using the method of inverse-variance weighting (IVW), with the error bar indicating the 95% confidence interval.

In the sensitivity analyses, we conducted heterogeneity statistics, horizontal pleiotropy assessment, and leave-one-out analysis. The results obtained from Cochran’s *Q* test indicated a lack of substantial heterogeneity among these distinct factors (Supplementary Table S2). Additionally, the MR-Egger regression intercept analysis (Supplementary Table S3) revealed no significant presence of horizontal pleiotropy in a particular direction. The leave-one-out analysis demonstrated the absence of any SNPs exerting a driving influence on the association between the gut microbiome and hypothyroidism (Figure 5). Additionally, we utilized the MR-PRESSO technique to detect aberrant SNPs and investigate whether the causal impact would be altered upon their exclusion. Ultimately, there was insufficient proof to back up the existence of horizontal pleiotropy in the relationship between these microorganisms and hypothyroidism (Supplementary Table S4).

## 4 Discussion

Our study used data from the MiBioGen consortium’s largest GWAS meta-analysis of the gut microbiome and pooled statistics on hypothyroidism published by the FinnGen Consortium R8. To assess the causal link between the gut microbiota and hypothyroidism, we conducted a two-sample MR analysis. Akkermansia, Butyrivibrio, Holdemania, and Ruminococcaceae UCG-011 were identified as protective factors for hypothyroidism, whereas Anaerostipes, Intestinimonas, and Ruminiclostridium5 were found to have negative effects on the condition.

Several observational studies have indicated a correlation between the gut microbiome and hypothyroidism. Veillonella, Paraprevotella, Neisseria, and Rheinheimera are some of the bacteria that have been previously identified as capable of differentiating primary hypothyroidism patients from healthy individuals (39). Comparing the radioactive iodine uptake in germ-free (GF) rats or rats treated with kanamycin to a control group, it was found that GF rats had decreased thyroid function (40). In 1996, a study additionally demonstrated that GF mice exhibited a 25% increase in TSH levels compared to mice possessing a regular gut microbiota (41). Additionally, scientists verified that a modified gut microbiota facilitated the development of hypothyroidism in mice through the process of fecal microbiota transplantation (42). These studies have shown a strong correlation between hypothyroidism and microflora dysbiosis. Nevertheless, the studies included varying sample sizes, ranging from a few dozen to several hundred, which might not accurately reflect the overall population. The current study included 18,340 samples of gut microbiome data from various ethnicities. In contrast, the dataset for hypothyroidism contains 86,169 samples from individuals with European backgrounds (26,342 individuals with the condition and 59,827 without), which enhances the inclusiveness and representativeness of this study.

Our study findings validated the correlation between Akkermansia and a decreased likelihood of hypothyroidism. Most cases of hypothyroidism were likely due to Hashimoto’s thyroiditis (43, 44). In a study of the diverse dispersion of gut microorganisms in individuals diagnosed with Hashimoto’s thyroiditis, it was observed that Akkermansia bacteria were more prevalent in both healthy individuals and those with Hashimoto’s thyroiditis with normal thyroid function, in contrast to the group of patients with Hashimoto’s with hypothyroidism (45). These results aligned with our findings. Derrien et al. were the first to describe the genus Akkermansia in 2004 (46), which is a helpful substance for gut health that strengthens the intestinal lining, boosts the mucus layer, and regulates the immune system. The link between Akkermansia and disease has been demonstrated in numerous human and animal studies. In a study conducted by Patial et al., the preventive effect of green tea catechins (GTC) on obesity-induced kidney injury was evaluated. The study revealed that catechins prevented gut ecological dysregulation in rats by modulating the PPARγ/CD36 pathway and promoting the growth of healthy microorganisms, such as *Akkermansia muciniphila* and *Lactobacillus reuteri* (47). This establishment of a gut-renal axis contributed to the prevention of obesity-induced kidney injury. Plovier et al. conducted a study where they purified the outer membrane protein of *A. muciniphila*, known as Amuc_1100. This protein was found to be stable even after pasteurization and has the ability to interact with toll-like receptor 2, thereby enhancing the function of the intestinal barrier (48). Additionally, it was observed that this protein alone contributes to some of the probiotic effects. It is important to note that Akkermansia not only has implications in metabolic diseases but also plays a role in the immune system response. Png et al. discovered that the abundance of Akkermansia is reduced in the intestinal mucosa of patients suffering from IBD (49). Given these beneficial properties and its prevalence across various life stages, Akkermansia is now considered a promising probiotic or live biotherapeutic product therapy, following Bifidobacterium (50). In exploring the pathogenesis of autoimmune thyroiditis, it has been found that increased intestinal permeability allows toxins, antigens, or bacterial metabolites to enter the bloodstream from the intestinal tract and promote disease. Therefore, the intestinal repair and immunomodulatory functions of Akkermansia may provide new insights into the prevention and treatment of hypothyroidism. However, randomized controlled trials are required to establish the beneficial effects of probiotics against hypothyroidism and their specific protective mechanisms.

Butyrivibrio bacteria are predominantly located in the rumen of ruminant animals, where they have a crucial function in breaking down plant fibers and producing butyric acid (51). The connection between Butyrivibrio and disease was established by observing a decrease in the proportion of Butyrivibrio species in the intestinal tracts of patients with Behcet’s disease. This finding suggests a potential association between Butyrivibrio and T-cell abnormalities resulting from changes in metabolites (52). However, the current body of literature pertaining to Butyrivibrio and hypothyroidism is limited, yet several studies have substantiated its capacity to generate short-chain fatty acids (SCFAs) and promote intestinal well-being (53). In the study of microorganisms that produce butyrate, the genus Butyrivibrio has been extensively researched. Butyrate is the preferred energy source for colonic cells and plays a crucial role in maintaining the colonic epithelium (54). SCFAs, including butyrate, also contribute to the regulation of tight junction proteins (TJPs), which are essential for the integrity of the epithelial barrier (55). The ability of Butyrivibrio to produce SCFAs and influence the intestinal environment may be a significant factor in hypothyroidism.

According to prior research, Holdemania has been linked to the development of specific illnesses, including delirium and Parkinson’s disease (56). Individuals diagnosed with hypothyroidism frequently encounter neuropsychiatric manifestations as a result of reduced thyroid hormone levels. In line with our study results, Liu et al. found a reduction in Holdemania among individuals with Hashimoto’s thyroiditis with hypothyroidism (45), indicating that Holdemania acts as a safeguard against hypothyroidism. Moreover, an excessive intake of alcohol has been associated with elevated levels of Holdemania in the gastrointestinal tract while causing a reduction in the concentration of butyric acid (57). Holdemania may potentially be involved in developing hypothyroidism by regulating the enterohepatic circulation of thyroid hormone. Previous studies have shown that the microbiota plays a role in enhancing the reabsorption of thyroid hormones in the enterohepatic circulation. This is achieved by breaking down sulfated glucuronide derivatives of iodothyronine through bacterial sulfate esterase or b-glucuronidase (58, 59). These findings could potentially shed light on the mechanisms underlying Holdemania’s involvement in hypothyroidism and offer valuable insights for future research.

Anaerostipes, a group of Gram-variable specialized anaerobes, are known for their production of acetic and butyric acids (60). Thi Phuong Nam Bui’s research findings indicate that a significant proportion of Anaerostipes species tested have the ability to convert inositol into propionate, potentially offering advantageous effects on health (61). Furthermore, various studies have established a correlation between Anaerostipes and specific diseases. An increased presence of Anaerostipes in the gut microbiota has been linked to an enhancement in glomerular filtration rate and a positive impact on renal function (62). Individuals with type 2 diabetes exhibit a decreased abundance of Anaerostipes in their gut compared to those in good health (63). A focused inquiry was conducted to explore the association between the gut microbiota and Graves’ disease (GD), uncovering that Anaerostipes was identified as a safeguarding element (64). Furthermore, Jiang’s study validated that individuals with GD exhibited reduced quantities of Anaerostipes in their gut microbiota in comparison to the control cohort (65). However, our findings indicated that Anaerostipes contributes to the development of hypothyroidism. These varying results are not surprising, as the composition of intestinal flora is influenced by multiple factors, including age, gender, race, medications, and dietary habits. Patients with GD are commonly treated with medications and radioactive iodine, which can have the side effect of causing hypothyroidism (66). It is known that hypothyroidism can lead to impaired gastrointestinal motility, creating conditions that favor the overgrowth of intestinal flora (67). However, the impact of these drugs on the composition of the intestinal flora and environment remains unknown. It is possible to hypothesize that the number and abundance of Anaerostipes may change during the recovery from the disease, and an increase in Anaerostipes could potentially contribute to the development of hypothyroidism. The alterations in the abundance of anaerobic bacteria in the gut may potentially regulate thyroid hormone levels, warranting further investigation in future studies.

Notably, most hypothyroidism cases arise from autoimmune dysfunction and are classified as chronic inflammatory disorders. The research conducted by Zhuang and Du et al. provided evidence suggesting a correlation between Intestinimonas and interleukin-4 levels, as well as the involvement of butyric acid produced by Intestinimonas in regulating T-cell differentiation and modifying interleukin-4 concentrations, leading to favorable anti-inflammatory outcomes (68, 69). Individuals diagnosed with Hashimoto’s thyroiditis but possessing normal thyroid function exhibited elevated levels of Intestinimonas in comparison to those diagnosed with both Hashimoto’s and hypothyroidism (17). Unexpectedly, our findings revealed a link between the presence of Intestinimonas and the development of hypothyroidism. This contradictory conclusion may be attributed to potential confounding variables in observational studies, thus emphasizing the need for rigorous randomized controlled trials. A decrease in the Ruminiclostridium5 abundance may be predictive of osteoarthritis (70), whereas studies on Ruminococcaceae UCG-011 remain limited.

As research on the gut microecology of thyroid disease advances, there is increasing evidence indicating that the gut microbiota plays a significant role in the progression of thyroid disease, either directly or indirectly. The microbiota and its metabolites may impact thyroid homeostasis through various pathways, including inducing immune-inflammatory responses, altering iodothyronine metabolism, and influencing the uptake of thyroid-associated micronutrients (71). One area of research interest is the impact of short-chain fatty acids (SCFAs) on thyroid function. These metabolites are produced by fermentation of dietary fiber by the intestinal flora, including acetic acid, propionic acid, and butyric acid. Maintaining intestinal homeostasis relies heavily on the composition and amount of the gut microbiota, which play a vital role in the production of short-chain fatty acids, particularly butyric acid (55, 72). SCFA-producing bacteria such as Akkermansia, Butyrivibrio, Holdemania, Anaerostipes, and Intestinimonas were identified in this study as part of the gut microbiota associated with hypothyroidism. SCFAs play a crucial role in the regulation of sodium/iodine symporter (NIS) expression in thyroid cells. Previous research has demonstrated that SCFA, particularly butyric acid, inhibits histone deacetylase (HDAC) and activates NIS re-expression in thyroid cancer cells, leading to redifferentiation and enhanced iodine uptake (73). Additionally, SCFAs like butyrate and propionate have been found to preserve the integrity of the intestinal barrier by influencing the expression of MUC2 mRNA levels (74). Consequently, a decrease in SCFA production results in an elevated release of lipopolysaccharide (LPS) into the bloodstream, as observed in patients with primary hypothyroidism in the study conducted by Zhao et al. (39). SCFA can also bind to one or more G protein-coupled receptors expressed by most immune cells to regulate their function (75, 76). On the other hand, an increase in butyrate levels has been found to have a direct relationship with the number of regulatory T (Treg) cells, which are essential agents in maintaining immune tolerance (77). SCFAs have a vital function in maintaining the equilibrium between T helper cell 17 (Th17) and Treg populations and are closely linked to the development of autoimmune disorders (78). Moreover, they are vital in strengthening tight intercellular junctions along with thyroid hormones, ensuring intestinal barrier integrity (79).

One of the leading causes of hypothyroidism is inadequate iodine intake (43). Several research studies have suggested that the composition of the gut bacteria affects the uptake and movement of essential minerals such as iodine, copper, iron, zinc, and selenium, along with the required enzyme activity for synthesizing thyroid hormones (80, 81). The conversion of thyroxine (T4) into active triiodothyronine (T3) or reverse T3 (rT3) heavily relies on iodothyronine-deiodinases (79). The intestinal wall exhibits deiodinase activity, which possibly increases the overall amount of T3 (82). Animal models have demonstrated that individuals deficient in certain microorganisms have a reduced ability to absorb elemental iodine through the intestine (58, 83). It has also been reported that the intestinal bacteria could take up deconjugated iodothyronine and even compete to bind thyroid hormone albumin (84). Therefore, probiotic or live biotherapeutic product and SCFAs are crucial factors in the development of hypothyroidism. Further studies are necessary to determine their specific roles in different pathway.

This study demonstrates several notable strengths. To establish a causal relationship between hypothyroidism and the gut microbiota, MR analysis was performed. This analysis effectively controlled for confounding factors and addressed the problem of reverse causation in causal inference. Furthermore, to guarantee the strength of our analytical method, we integrated genetic diversity in the gut microbiota by conducting a thorough GWAS meta-analysis. In addition, we utilized MR-PRESSO and MR-Egger regression intercept tests to detect and remove any possible horizontal pleiotropy. We implemented a two-sample MR design with distinct exposures and summary-level data to reduce bias.

However, when analyzing the findings, it is crucial to consider particular constraints inherent in this research. Unfortunately, the provided dataset only contained data at the genus level, which limited our ability to investigate the correlation between the gut microbiota and hypothyroidism at the species level. To perform sensitivity analyses and test for horizontal pleiotropy, a greater range of genetic variation would be required as an instrumental variable. Due to the limited size of the gut flora samples, the SNPs utilized in the study did not reach the standard GWAS significance threshold (*p* < 5 × 10^−8^). The primary focus of the GWAS meta-analysis performed using the gut microbiota data was individuals of European descent. Nonetheless, it is crucial to recognize the possible existence of confounding population stratification, which could restrict the applicability of the results to populations outside Europe. To improve the applicability and inclusiveness of future MR research investigating the causal link between the gut microbiota and hypothyroidism, it is advisable to incorporate a wide range of populations, including individuals of European and non-European ethnicities.

## 5 Conclusion

According to a study using two-sample MR, there appears to be a causal link between the gut microbiota and hypothyroidism. The results of this study demonstrated that Akkermansia might act as a protective factor against hypothyroidism, opening up new opportunities for the prevention and treatment of hypothyroidism. However, additional randomized controlled experiments are necessary to determine the precise mechanism. Additionally, while reverse MR did not provide evidence of hypothyroidism impacting the intestinal flora, it is still possible that the condition may affect the microecology of the intestines. Confirming this notion would require rigorous studies.

## Data availability statement

The original contributions presented in the study are included in the article/Supplementary material, further inquiries can be directed to the corresponding author.

## Ethics statement

The research conducted in this study was based on published studies and consortia, which provided publicly available summary statistics. The corresponding ethical review board approved all the original studies, and the participants had provided informed consent. Furthermore, no individual-level data was used in the study, which meant that no new ethical review board approval was required.

## Author contributions

CS: Data curation, Formal analysis, Visualization, Writing – original draft. JC: Data curation, Resources, Writing – review & editing. SH: Writing – review & editing, Supervision. YiZ: Writing – review & editing. YaZ: Writing – review & editing. LY: Writing – review & editing.
