# Supplementary figures and images for: Cross-talk between the gut microbiota and hypothyroidism: a bidirectional two-sample Mendelian randomization study
Source: Front Nutr. 2024 Mar 18;11:1286593. doi: 10.3389/fnut.2024.1286593 (PMC10982496; doi:10.3389/fnut.2024.1286593)

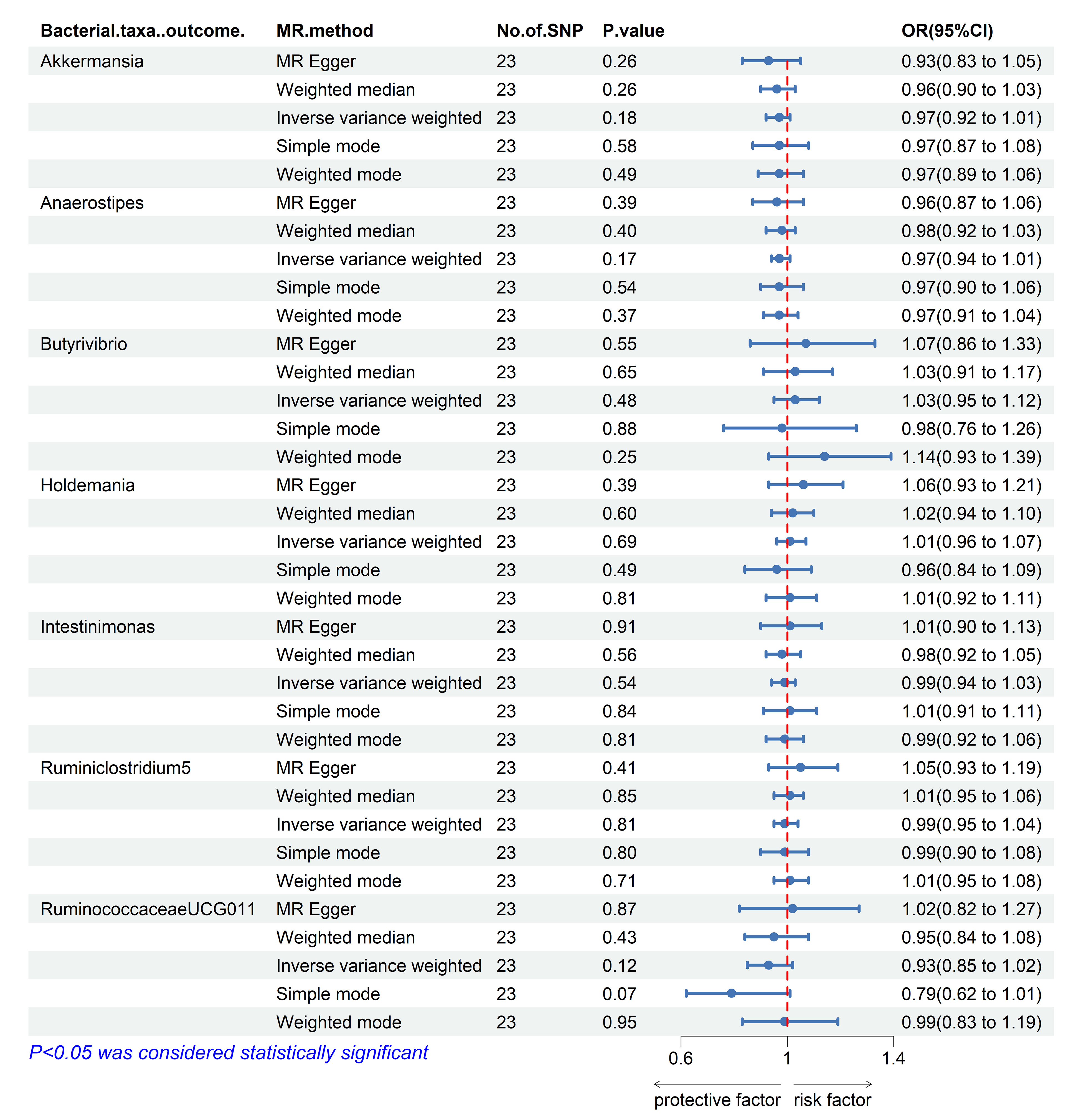

Supplement: Supplementary file 1 [file Image_1.JPEG]
